# With the Red Cross in Palestine

**Published:** 1918-05-25

**Authors:** 


### With the Red Cross in Palestine.

### DR. ALEXANDER GRANVILLE'S REPORT.

The campaign in Palestine, which has necessarily extended the work of the Red Cross, is the subject of a report by Dr. Alexander Granville, British Red Cross Commissioner at Alexandria, to Sir Arthur Stanley, Chairman of the Joint War Committee. The report is the outcome of a visit of inspection to the hospitals and medical units on the Palestine Front. In the course of his remarks Dr. Granville says: "Our work on that Front has now become one of the most important features of our activities. We have a splendidly situated base store at Kantara, which is fed from here (Alexandria), and which in turn feeds our depôts at Port Said, Ismailia, and Suez, as well as the hospital and medical units from Kantara up to Rafa. In addition, the Kantara base store keeps our advanced stores at Jerusalem, Jaffa, Ludd, and Junction Station supplied with whatever they want. This Kantara base store has been invaluable to us, as it has meant that much time has been saved in getting stores through to our advanced stores in Palestine.

"In Jerusalem we have a particularly fine store in a building which has been generously placed at our disposal by the Mother Superior of the Marie Reparatrice Convent. In Jaffa we have been given the use of a very well-situated house for our stores and officers. At Ludd and Junction our advanced stores are under canvas, and are placed in most convenient places. As soon as the necessity arises we will establish another store at Gaza, from which we shall have to supply two big general hospitals and three stationary hospitals with whatever Red Cross stores they require.

"Our Red Cross invalid kitchens at El Arish are a great success. We have two excellent cooks running these kitchens. I hope to be able to establish very soon another two Red Cross invalid kitchens on this Front.

### An Interesting Visit.

"The Duke of Connaught, I understand, was much impressed by his visit to the great Red Cross Hospital of Montazah with its 1,500 beds, and by the good use which we have made of this charming place, and was well pleased with all that he saw. It is interesting to note that 42,300 men have passed through this hospital since it was opened in 1915, and have greatly benefited by the period of convalescence amid such beautiful surroundings."

